# Characterization of the Intestinal Fungal Microbiome in HIV and HCV Mono-Infected or Co-Infected Patients

**DOI:** 10.3390/v14081811

**Published:** 2022-08-18

**Authors:** Yue Yin, Maermaer Tuohutaerbieke, Chengjie Feng, Xinjie Li, Yuqi Zhang, Qiang Xu, Jing Tu, Ence Yang, Qinghua Zou, Tao Shen

**Affiliations:** 1 Department of Microbiology and Infectious Disease Center, School of Basic Medical Sciences, Peking University, Beijing 100191, China; 2 Department of Microbiology, School of Basic Medical Sciences, Peking University Health Science Center, Beijing 100191, China

**Keywords:** human immunodeficiency virus, hepatitis C virus, intestinal fungal dysbiosis, CD4 + T cells, ALT, opportunistic pathogens

## Abstract

Intestinal mycobiome dysbiosis plays an important role in the advancement of HIV- and HCV-infected patients. Co-infection with HCV is an important risk factor for exacerbating immune activation in HIV-infected patients, and gut fungal microbial dysbiosis plays an important role. However, no systematic study has been conducted on the intestinal fungal microbiome of HIV/HCV co-infected patients to date. Patients infected with HIV and HCV, either alone or in combination, and healthy volunteers were included. Stool samples were collected for fungal ITS sequencing and for further mycobiome statistical analysis. We found that the abundance of fungal species significantly decreased in the HIV/HCV co-infection group compared to in the healthy control group, while no significant differences were found in the mono-infection groups. Low-CD4 + T-cell patients in the HIV group and high-ALT-level patients in the HCV group were discovered to have a more chaotic fungal community. Furthermore, the opportunistic pathogenic fungal profiles and fungal inter-correlations in the co-infection group became less characteristic but more complicated than those in the mono-infection groups. Intestinal fungal dysregulation occurs in HIV- and HCV-infected patients, and this dysregulation is further complicated in HIV/HCV co-infected patients.

## 1. Introduction

Human immunodeficiency virus type 1 (HIV-1) has become a worldwide threat to human health. In addition to the continuous replication of HIV itself, intestinal microecological changes and co-infection with other chronic viral infections, such as HCV, are considered to be the key reasons for the persistence of immune activation in HIV-infected patients. Since 2012, several researchers have successively proposed that fungal translocation is associated with immune activation and systemic inflammation in HIV-infected patients undergoing ART treatment [1,2,3,4,5,6]. Fungi contribute significantly to opportunistic infections in people living with HIV (PLWH), especially in immunocompromised patients with low CD4 + T-cell counts [7]. As the immune status of patients declines, opportunistic infections will occur and eventually lead to death. Deaths due to invasive fungal infections in HIV-infected patients account for 50% of HIV-related mortality globally [8]. Some findings have led researchers to put forward the hypothesis that the intestinal fungal microbiome may be responsible for fungal infection [9,10,11,12]. Fungal communities, which consist of nearly 0.1% of the total microbes in the gut [13,14], have begun to attract more widespread attention.

However, there are only a few studies regarding intestinal fungi in HIV-infected patients that have been conducted thus far. Some researchers have found that compared to healthy controls, fungi show a higher prevalence in HIV-diarrhea patients, of which C. parvum, C. difficile, and C. albicans were the most representative species [15,16,17,18,19]. A negative correlation between diarrhea and candidiasis in AIDS patients has also been reported [20,21]. Hepatitis C virus (HCV) infection, a major risk factor for cirrhosis, hepatocellular carcinoma, and death [22,23], has been confirmed as an important cause of morbidity and mortality in people who are co-infected with HIV [24]. The clearance rate of HCV was decreased due to immunodeficiency resulting from HIV infection [25]. HIV also interacts with HCV through many pathways, such as through directly infecting hepatocytes [26,27,28], promoting HCV replication [29,30,31], and impairing the anti-fibrotic activity of NK cells [32,33,34].

As there have been limited studies on intestinal fungal dysbiosis in HIV mono-infected patients, HCV mono-infected patients, and HIV/HCV co-infected patients, in this study, we characterized the intestinal mycobiome communities in patients from these groups, aiming to identify specific mycobiome lineages that may play important roles in the development of disease and trying to identify important intestinal fungi in HIV/HCV co-infected patients to better understand the disease status of co-infected patients for treatment.

## 2. Materials and Methods

### 2.1. Study Cohort

In this study, 87 patients infected with HIV and/or HCV and 22 healthy controls matched by age and BMI were recruited. Both the HIV and HIV/HCV co-infected patients had been infected for an average of 22 years and had received cART for an average of 13 years. All of the HIV patients had been infected through blood transmission. The diagnosis of HIV-infected individuals was verified using PCR and HIV-1 antibody tests, and the diagnosis of HCV infection was based on serologic markers, virological markers, liver function tests, and biochemical assays. HIV mono-infected patients and HIV/HCV co-infected individuals had all been treated with two nucleoside reverse transcriptase inhibitors (NRTIs) and nonnucleoside reverse transcriptase inhibitors (NNRTIs) for more than 10 years, while no antiviral treatment had been taken in the HCV mono-infection group.

The exclusion criteria were age >72 years or <40 years, the use of antibiotics or immunosuppressive drugs or antifungal drugs within the past 1 month, a history of gastrointestinal disease or gastrointestinal surgery, and evidence of hepatitis A or B virus infection or other chronic diseases. Clinical and demographic data were obtained by performing standardized subject interviews and medical record reviews.

### 2.2. Sample Collection

Fecal samples were collected from the subjects, with each individual sampled once. Each sample was immediately placed in a sterile plastic container and stored at −80 °C before being processed. DNA was extracted using the QIA amp DNA Stool Mini Kit (QIAGEN, Hilden, Germany) according to the instructions. DNA quantification and purity were assessed using a NanoDrop ND-1000 spectrophotometer (Thermo Electron Corporation, Waltham, MA, USA).

### 2.3. PCR Amplification

The primers 5′-CTTGGTCATTTAGAGGAAGTAA-3′ and 5′-GCTGCGTTCTTCATCGATGC-3′ were used for ITS rDNA gene amplification. Oligonucleotide primers were synthesized by the Shanghai Sangon Biotechnology Limited Company (Shanghai, China). The PCR products were extracted from a 2% agarose gel and purified using the AxyPrep DNA Gel Extraction Kit (Axygen Biosciences, Union City, CA, USA) according to the manufacturer’s instructions and were quantified using a Quantus™ Fluorometer (Promega, Madison, WI, USA).

### 2.4. Illumina MiSeq Sequencing

Purified amplicons were pooled in equimolar amounts and were paired-end sequenced on an Illumina MiSeq PE300 platform (Illumina, San Diego, CA, USA) according to the standard protocols by Majorbio Bio-Pharm Technology Co. Ltd. (Shanghai, China). The raw ITS gene sequencing reads were demultiplexed, quality-filtered by fastp [35] version 0.20.0 (https://github.com/OpenGene/fastp, version 0.20.0), and merged using FLASH [36] version 1.2.7 (http://www.cbcb.umd.edu/software/flash, version 1.2.7). Operational taxonomic units (OTUs) with a 97% similarity cutoff were clustered using UPARSE [37] version 7.0.1090 (http://drive5.com/uparse/, version 7.1), and chimera identification sequences were identified and removed. The RDP classifier [38] known as the Bayesian algorithm (http://rdp.cme.msu.edu/, version 2.2) was used in combination with the Unite [39] (Release 8.0 http://unite.ut.ee/index.php) database for taxonomic analysis at a 70% cutoff confidence level for each taxonomic level: domain, kingdom, phylum, class, order, family, genus, and species, to calculate the community species composition of each sample.

### 2.5. Statistical Analysis

Categorical variables were analyzed using the chi-square test. Comparisons between three or more groups were analyzed by one-way ANOVA or the Kruskal–Wallis test. Comparisons between two groups were performed with an unpaired *t*-test or the Mann–Whitney U test. Relationships between variables were analyzed by Spearman’s rank correlation coefficient. Statistical analysis was performed using SPSS 25.0 (SPSS^®^ Inc., Chicago, IL, USA) and GraphPad Prism 7.00 (GraphPad Prism^®^, La Jolla, CA, USA). Alpha diversity and beta diversity were assessed using the R statistical software package (version 3.6.1; www.r-project.org). The effect size of the linear discriminatory analysis (LDA) was an LDA score > 2.5 and a *p* value < 0.05. Spearman correlations between fungi were calculated using Origin software (version 2021b) with a cutoff of 0.5 and *p* value < 0.05 and were plotted as a heatmap. A Venn diagram was used to display the numbers of common and unique OTUs and the shared OTUs among different samples.

## 3. Results

### 3.1. Participant Characteristics

In total, 18 HIV mono-infected patients, 40 HCV mono-infected patients, 29 HIV/HCV co-infected patients, and 22 healthy individuals were enrolled. Their basic information can be found in Table S1. The median CD4 + T-cell count of the HIV mono-infected group was 484 cells/μL (IQR 201-694) and included the counts from four severely immunocompromised individuals with CD4 + T-cell counts below 200 cells/μL. The median CD4 + T-cell count of the HIV/HCV co-infected group was 514 cells/μL (IQR 463-629), with only one patient whose CD4 + T-cell counts were below 200 cells/μL. Although the number of immunocompromised individuals was lower in the HIV/HCV co-infected group (*p* = 0.0424), the CD4 + T-cell counts showed no differences between the two groups. Liver enzyme indicators in serum were significantly higher in the HCV mono-infected patients than in the healthy controls, and co-infection with HIV may further exacerbate abnormal liver function, as reflected by a further increase in the glutamate transaminase, glutamic oxaloacetic transaminase, and gamma-glutamyl transpeptidase levels in HIV/HCV co-infected patients compared to in HCV mono-infected patients.

### 3.2. Taxonomy and Alpha Diversity of the Four Groups

**Figure 1:**
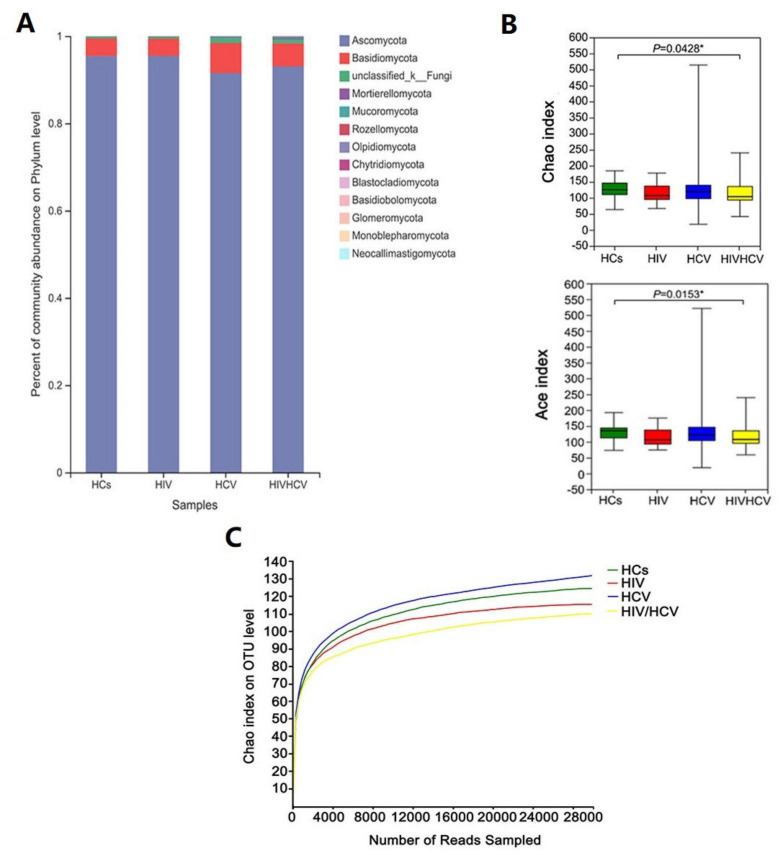
Taxonomy and alpha diversity of the four groups. (**A**)The community histograms at phylum level. (**B**) Chao and ACE diversity index of the four groups. (**C**) Rarefaction curves of Chao index at out level. Clustering was performed by Uparse 7.0.1090. Each curve represents the average of all repeats (±standard deviation) for each group of samples (HCs, HIV, HCV, and HIV/HCV). * *p* < 0.05, determined by unpaired Student’s *t*-test.

The basic information obtained from the sequencing reads and operational taxonomic units (OTUs) for each sample can be found in Table S2. At the phylum level, the OTUs were assigned to twelve known fungal phyla (Figure 1A). The predominant phylum was Ascomycota (93.36%, 51.64–99.99%), followed by Basidiomycota (5.42%, 0.01–46.96%), and Mortierellomycota and Mucoromycota (0.13%, 0–1.26%). Ascomycota and Basidiomycota were prevalent in all of the tested samples.

There were no significant differences in the Shannon and Simpson indexes among the four groups (Table S3), and obvious changes in the diversity of the intestinal fungal community were not observed after disease onset. However, compared to normal people, the ACE index and Chao index scores of the HIV/HCV co-infected group were significantly lower (*p* = 0.01531, *p* = 0.04281), indicating that the abundance of the intestinal fungal community in HIV/HCV co-infected patients was reduced (Figure 1B), probably due to highly competitive resident microorganisms [40].

The rarefaction curve results based on the Chao index are shown in Figure 1C. The rarefaction curve began to flatten at 4000, indicating that the depth of sequencing was sufficient to reliably describe the fungal communities of the participants.

### 3.3. HIV Mono-Infected Patients Had an Altered Gut Mycobiome Compared to the HC Group

**Figure 2:**
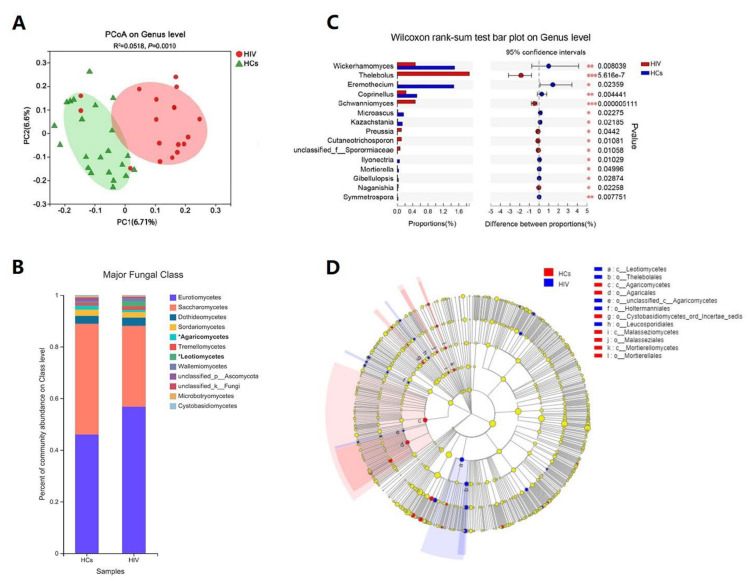
Characteristics of intestinal fungal spectrum in the HIV mono-infected patients and healthy control group. (**A**) Beta diversity (PCoA-based unweighted UniFrac distance matrix) between the two groups. (**B**) The changes in the intestinal fungal spectrum in the HIV group compared to the healthy controls at class level. (Classes differing significantly are marked with ‘*’.) (**C**,**D**) present the differences in the HIV mono-infected patients and healthy controls through the Wilcox rank-sum test and LEfSe cladogram (LDA = 2.5), respectively.

Beta diversity analysis showed that the HIV mono-infected intestinal mycobiome was distinct from that of the healthy individuals (R^2^ = 0.0518, *p* = 0.001) (Figure 2A). The community histograms showed an elevated level of class Eurotiomycetes among the HIV mono-infected patients compared to the HC group, while the level of Saccharomycetes declined (Figure 2B). A detailed histogram of the genus level (Figure S1) showed that in the HIV mono-infected group, Aspergillus was the most abundant genus (49.92%), while in the healthy controls, the most abundant fungal genus was Candida (38.31%). The Wilcox rank-sum test and linear discriminatory analysis (LDA) effect size (LEfSe [41]) analysis showed the fungal genera that were differentially abundant in the two groups (Figure 2C,D). Of note, the species that was significantly enriched in the HIV mono-infected group (*p* < 0.05) was the class Leotiomycetes (bold in Figure 2B), which was elevated for the members Thelebolales, Thelebolaceae, and Thlebolus, which are usually isolated from freshwater, salt lake soils, and sponges [42] and were detected in human feces in our study for the first time. However, the class Agaricomycetes (bold in Figure 2B), ranking fifth among the normal fungal classes in the HCs (Figure 2B), significantly decreased in the HIV group. Schwanniomyces, a fungus that is rare in humans, was also enriched in the HIV mono-infected group (Figure 2C).

### 3.4. Immune Status Significantly Impacts the Gut Mycobiome of HIV Mono-Infected Patients

**Figure 3:**
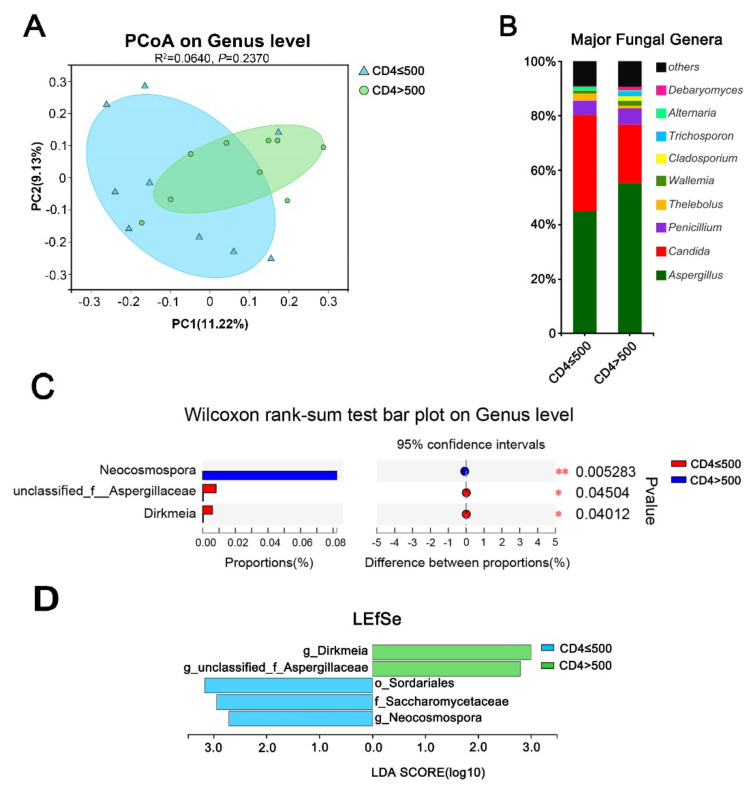
Effect of level of CD4 + T cells on the intestinal fungal profile of HIV mono-infected patients. (**A**) Beta diversity (PCoA-based unweighted UniFrac distance matrix) between the two groups. (**B**) Community histograms of intestinal fungal genus levels between the low-CD4 + T group (CD4 + T ≤ 500/μL) and the high-CD4 + T group (CD4 + T > 500/μL) within the HIV mono-infected group. (**C**) Bar plot result of Wilcox rank-sum difference analysis. (**D**) LEfSe discriminant histograms between the two groups (LDA = 2.5).

Furthermore, to investigate the effect of immune status on the intestinal fungal profile in HIV mono-infected patients, we studied the relationship between CD4 + T-cell counts and the gut mycobiome in the HIV mono-infected participants. As shown in Figure 3, patients with low CD4 + T-cell counts and patients with high CD4 + T-cell counts were found to have different fungal community characteristics. However, the PCoA analysis showed that the distribution of the samples from the HIV and HC groups was slightly different (Figure 3A). Similar to the results obtained when the HIV group was compared to the HC group, a community of bar plot analyses conducted at the class level (Figure S2) showed that histogram differences were mainly observed in the proportion of Eurotiomycetes, which was significantly decreased in the low-CD4 + T-cell group compared to in the high-CD4 + T-cell group, and in the levels of Saccharomycetes, which were significantly increased in the low-CD4 + T-cell group. Looking into the histogram *composition* at the genus level in depth, what is worth noticing is a sharp increase in *Candida* in the low-CD4 + T-cell group, indicating a high risk of opportunistic infection. Through further analysis, there were several genera worth noting (Figure 3C,D). More specifically, patients in the high-CD4 + T-cell group contained more g__ unclassified_f__Aspergillaceae and Dirkmeia, while Sordariales, Saccharomycetaceae, and Neocosmospora were mainly enriched in the low-CD4 + T-cell group, that is to say, the immunocompromised HIV patients.

### 3.5. HCV Mono-Infected Individuals Had an Altered Gut Mycobiome Compared to the HC Group

**Figure 4:**
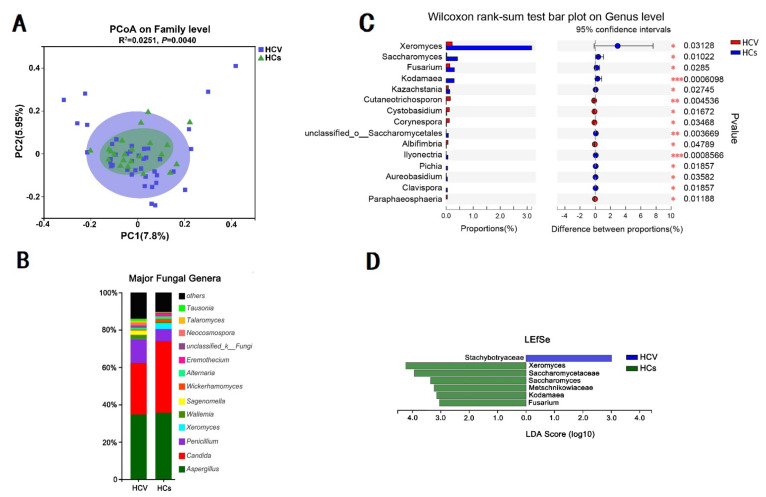
Characteristics of intestinal fungal spectrum in HCV mono-infected patients and in the healthy control group. (**A**) Beta diversity (PCoA-based unweighted UniFrac distance matrix) between the two groups. (**B**) Changes in the intestinal fungal spectrum in patients infected with HCV compared to healthy controls at the genus level. (**C**,**D**) present the differences between the HCV mono-infected group and the healthy controls through the Wilcox rank-sum test and LEfSe histograms (LDA = 3.0), respectively.

PCoA analysis showed that compared to the HIV mono-infection and HC group (Figure 2A), there were more similarities but fewer differences between the HCV mono-infection and HC group (Figure 4A). However, detailed histogram analysis at the genus level revealed a sharp decrease in Penicillium along with an elevated level of Candida in the HCV mono-infected group compared to in the healthy group (Figure 4B). Apart from these two genera, Wilcox analysis also illustrated that the HCV group had a decreased abundance of the genera Xeromyces and Saccharromyces (Figure 4C). LEfSe analysis revealed that a specific family, Stachybotryaceae, which was not previously found in humans, was significantly enriched in the HCV mono-infected group (Figure 4D).

### 3.6. Effect of Alanine Aminotransferase on the Intestinal Fungal Profile of HCV Mono-Infected Patients

**Figure 5:**
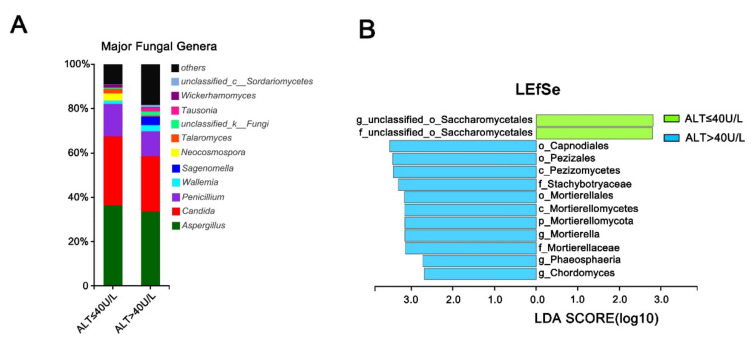
Effect of ALT level on the intestinal fungal profile of HCV mono-infected patients. (**A**) The community histograms of intestinal fungal genus levels between the ALT normal group (ALT ≤ 40 U/L) and the high-ALT-level group (ALT > 40 U/L) within the HCV mono-infected group; (**B**) LEfSe discriminant histograms between the two groups (LDA = 2.5).

We divided the HCV mono-infected individuals into two groups based on alanine aminotransferase levels and compared the fungal composition characteristics between the low-ALT-level group and the high-ALT-level group. As is shown in the bar chart in Figure 5A, the abundance of some dominant species in the normal ALT group clearly decreased in the high-ALT group. LEfSe analysis revealed specific fungal characteristics in the HCV mono-infected patients with high ALT levels (Figure 5B) and who contained large numbers of the phylum Mortierellomycota; the orders Capnodiales, Pezizales, and Mortierellales; the families Stachybotryaceae and Mortierellaceae, and the genera Mortierella, Phaeosphaeria, and Chordomyces. Most of the fungi mentioned above are endophytic fungi that are commonly found in plants or soil.

### 3.7. The Intestinal Fungal Profile Was Altered When HIV-Infected Patients Were Co-Infected with HCV

**Figure 6:**
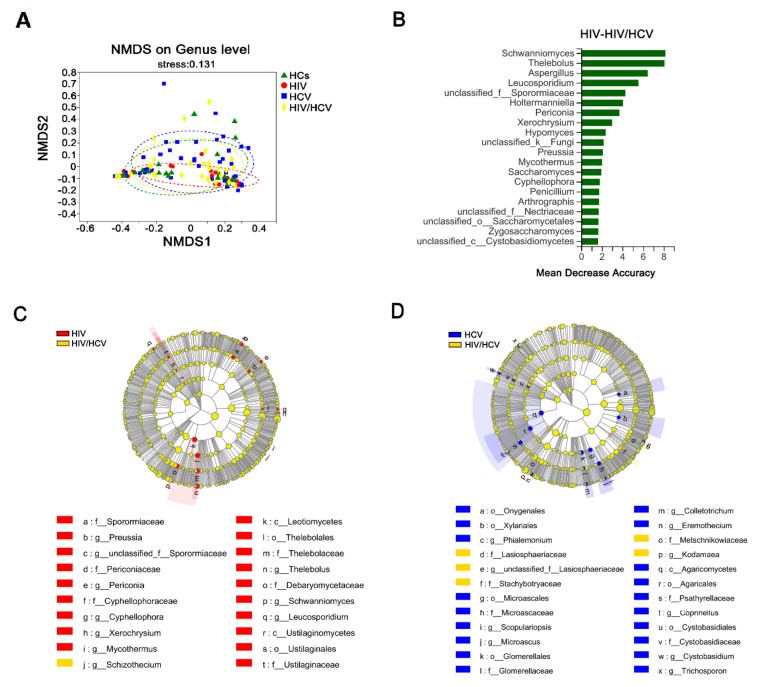
Differences in the gut mycobiome between mono-infected groups co-infected group. (**A**) Fungal β-diversity (NMDS-based Bray–Curtis distance matrix) between the four groups at genus level. (**B**) Random forest graph showing the 20 most predictive fungal genera between HIV mono-infection and HIV/HCV co-infection patients. (**C**,**D**) show the LEfSe discriminant cladograms between the HIV or HCV mono-infection and co-infection groups (LDA= 2.5).

We compared the fungal microbiome of the HIV/HCV co-infection group with that of the mono-infection group and unexpectedly found that the co-infection group was more similar to the HC group than the HIV mono-infection group, which is clearly shown in the NMDS analysis (Figure 6A). Based on the comparison of the HIV mono-infection vs. HIV/HCV co-infection groups (Figure 6C), we found that compared to the mono-infection group, the HIV/HCV co-infection group had fewer unique fungal species, while more unique fungal genera characterized the HIV mono-infection group rather than the co-infection group. Through further analysis, we found that the co-infection group lost many of the unique fungal profiles found in the HIV mono-infection group, such as the class of Leotiomycetes and genus of Preussia, both of which were enriched in the HIV mono-infection group compared to the HC group (see Figure 2C,D). When comparing the co-infected group with the HCV mono-infected group, we also discovered that some of the feature fungal profiles of the HCV group were lost after co-infection (Figure 6D). Coinciding with the decrease in the fungal abundance index in the co-infection group (Figure 1B, Chao and ACE index), the elimination of certain fungal profiles probably indicates the decline of fungal diversity in co-infection groups, which is probably due to the fierce competition between species (which will be discussed in the next section), leading to a much more chaotic intestinal microbiome. In addition, the co-infection group also presented an elevated level of Schizothecium compared to the HIV mono-infection group.

The random forest model also revealed the most predictive fungal genera that differed between the HIV group and the HIV/HCV co-infection group (Figure 6B), and the top two genera both belonged to the class Leotiomycetes mentioned above.

### 3.8. Fungal Inter-Correlations Differed between the Mono-Infection and Co-Infection Groups

**Figure 7:**
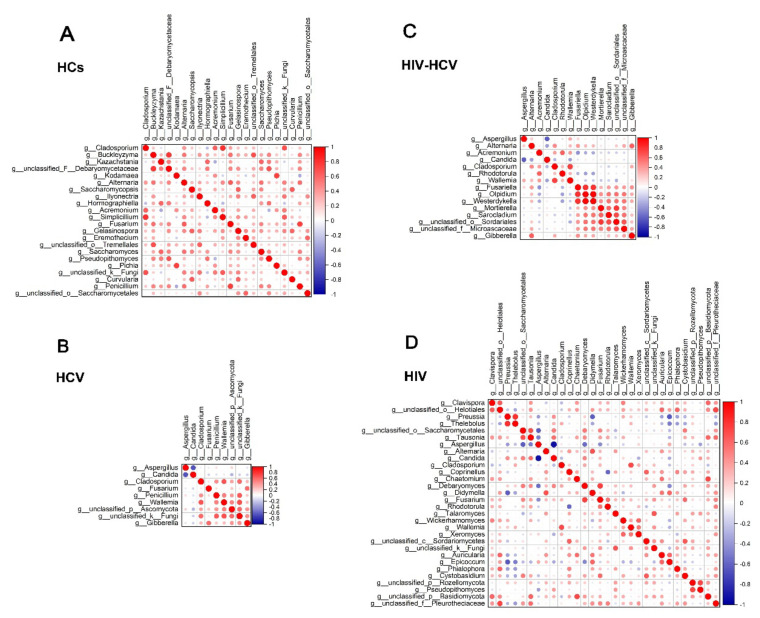
Correlation coefficients of mycobiome abundance in healthy controls (**A**), HCV mono-infected patients (**B**), HIV/HCV co-infected patients (**C**), and HIV mono-infected patients (**D**). Correlation of the mycobiome was determined using R statistical computing software (Spearman’s correlation and two-tailed probability of t for each correlation) for the four groups. Red: positive correlation; Blue: negative correlation; diameter of circles represents the absolute value of correlation for each pair of the fungi–fungi matrix. (Spearman rho > 0.6; *p*-value < 0.05).

Finally, we conducted correlation analysis using R statistical computing software to determine the correlation between different members of the mycobiome, and the correlation coefficients of each fungal pair are listed in Table S4. The correlation heatmap in Figure 7 shows the correlated fungal pairs whose correlation coefficients were greater than 0.6. We found 17 positively correlated fungal pairs (Figure 7A) in samples from healthy participants. Among them, the most positively correlated fungal pair was Cladosporium-Simplicillium (R = 0.8031, *p* < 0.0001).

Seven significantly correlated fungal pairs were detected in the HCV mono-infected group (Figure 7B), and the one with the strongest positive correlation was Wallemia-g__unclassified_k__Fungi (R = 0.6988, *p* < 0.0001). A negative correlation was also observed between Aspergillus and Candida (R = −0.6786, *p* < 0.0001).

There were 25 significantly correlated fungal pairs in the HIV mono-infected group (Figure 7D), which were much more complicated than those in the other groups. The most negatively correlated pair was Candida–Aspergillus (R = −0.9360, *p* < 0.0001).

For the HIV/HCV co-infected group, we found 11 significantly correlated fungal pairs, which was more than the number of correlated fungal pairs in the HCV group but less than in the HIV group (Figure 7C). The most negatively correlated fungal pair was Aspergillus–Candida (R = −0.6207, *p* = 0.0003). Apart from the combined features of the mono-infection groups, there were also some novel pairs that were absent in the mono-infection groups, such as the negative correlation between Alternania and Candida, Rhodotorula and Olpidium, and Acremonium and Fusariella, and some positive reaction groups, such as the positive correlation square in the right lower corner of Figure 7C.

In addition, we could see that there were many more negative correlations in the infection groups than in the healthy controls. The most typical pair was the strong negative correlation between Candida and Aspergillus in all three infection groups, which was absent in the HC group.

## 4. Discussion

In the healthy human gastrointestinal tract, the most common fungal taxa are yeasts such as *Candida*, *Saccharomyces*, and *Malassezia* and filamentous fungi such as *Aspergillus*, *Cladosporium*, and *Penicillium*. Collectively, these fungal constituents represent core mycobiota species [43]. The fungal microbiome of the HIV mono-infection group was significantly different from that of the HC group, and the most abundant fungal family found in the HIV mono-infection group was different from that of the HC group. In addition, some opportunistic fungi, such as *Neocosmospora rubeola*, were clearly enriched in low-CD4 + T-cell patients with HIV infection compared to in patients with normal CD4 + T cell levels. Members of the genus *Neocosmospora* have been reported to cause lung infections in liver transplant patients [44] and to encompass highly prevalent and aggressive human and animal fungal pathogens [45], indicating that the decrease in T cells exposes the patient to a state of high-risk infection. Consistent with this, some researchers have compared the mycobiome of patients receiving ART treatment, finding that some fungi are closely correlated with virus load (VL) and CD4 + T-cell count [46]. However, there were more similarities and fewer differences between the HCV mono-infection and HC groups. However, the special family *Stachybotryacea*, which has not been previously reported in humans, was significantly enriched in the HCV mono-infection group. The mechanism leading to this remains unclear. Further analysis of the correlation between ALT and the fungal profile revealed that the fungal profile of the high-ALT group was more diversified, and some of those enriched fungi (such as *Mortierella* and *Sagenomella*) have been reported to cause fungal infections in humans [47,48]. We are not confident enough to state the cause of the effect (high ALT and special fungal profile), but we can infer that patients with higher levels of ALT may have a greater risk of opportunistic fungal infections.

Compared to the HIV/HCV mono-infection groups, some of the fungi enriched in the co-infection group were not discovered through mono-infection vs. HC comparison, such as the plant-originated fungi *Lasiosphaeriaceae* and *Metschnikowiaceae*, the human-infecting fungus *Kodamaea ohmeri,* and the opportunistic fungus *Curvularia hominis*. Notably, the co-infection group lost many unique fungal profiles of the HIV group, such as the class *Leotiomycetes* and genus *Preussia*, both of which were enriched in the HIV group when compared to the HC group, yet diminished in the co-infection group for some reason, which indicates that as the co-infection of the two viruses takes place, the fungal profile becomes less characterized and more complicated. What comes along with the presence of some novel fungal genera is many unique fungal inter-correlations, which may be positive or negative.

Fungal inter-correlation analysis also presents some interesting outcomes. There are some similarities between the four groups. For example, the positive correlation between *Penicillium citrinum* and *Aspergillus minisclerotigenes* was present in all of the groups except the co-infection group, and the correlation coefficients showed no significant difference, indicating that alternation of the gut environment does not have any impact on the correlation of the two fungi. However, the positive correlation between *Aspergillus penicillioides* and *Aspergillus cibarius* became stronger in the co-infection group than in the HCV mono-infection group. We can infer that this might be the result of the decrease in immunity caused by HIV infection. Additionally, taking an overall glance at the inter-correlation heatmap, we found that there were more negative correlations in the mono/co-infection groups than in the healthy group, which may indicate that infection causes fierce competition in the fungal microbiome in the patient’s gut, speeding up the process of natural selection. The most representative pair was *Candida* and *Aspergillus*, whose correlation coefficient reached −0.9360 in the infection groups but showed no significant relationship in the HC group. Taking into consideration the fact that these two fungi are the top two predominant fungi in all four groups but that their relative abundance is different in the HC group and the infected group, we can infer that mutual inhibition results in the alternation of the predominant fungus, participating in the advancement of the disease.

Exploring the correlation between intestinal fungi has good clinical application prospects. For example, the antagonism between the harmless fungus and the opportunistic pathogenic *Candida* has shown extremely high clinical application value. However, this method is not necessarily a one-time-for-all approach. Antifungal treatment administered by artificially adding fungi to the mycobiome (such as through probiotic supplements) can avoid the tolerance caused by the abuse of antifungal drugs, but it also destroys the original intestinal microenvironment. The long-term use of this method in intervening in vivo fungal infection is worthy of further exploration.

## 5. Conclusions

This study preliminarily explored the characteristics of the intestinal fungal community in HIV- and HCV-alone and co-infected conditions. The fungal abundance indexes of the infection groups were lower than those of the healthy controls. The fungal microbiomes of the HIV and HCV mono-infection groups were significantly different from those of the HC group, and some opportunistic fungi were clearly enriched in low-CD4 + T-cell patients and in high-ALT-level patients. The fungal profile of the HIV/HCV co-infection group was less characteristic but more complicated than that of the mono-infection groups. As for the inter-correlation of fungi, apart from some similarities, there were more negative correlations in the mono/co-infection groups than in the healthy group. Infection also led to some significant changes in the inter-correlation of certain fungal pairs.

## 6. Strengths and Limitations

The HIV patients in our research cohort were all infected through blood transmission. They had no history of drug abuse or other unhealthy lifestyles and were similar to healthy people in terms of most of their living habits. From this perspective, this population provides a consistent background for studying the effects of HIV and/or HCV on differences in gut fungal profiles. At present, there are few studies on the characteristics of the intestinal fungal community in HIV-infected patients, and the changes in the intestinal fungal composition of patients with disease progression are still unclear. Therefore, although our research presented a large number of results, as a cross-sectional study, our study still lacks the support of previous research and follow-up data.

## Data Availability

The data used to support the findings in this study are available from the corresponding authors upon request.

## Supplementary Materials

The following supporting information can be downloaded at https://www.mdpi.com/article/10.3390/v14081811/s1, Figure S1: Histograms between HIV group and HC group of the genus level; Figure S2: Histograms between low-CD4+ group and normal CD4+ group of the class level; Table S1: Demographic and clinical features of 109 study participants; Table S2: Characteristics of sample sequencing length; Table S3: Comparison of fungal alpha diversity indexes among four groups; Table S4: Correlation among gut fungi in study participants.
