# Supplementary material for: Characterization of the Intestinal Fungal Microbiome in HIV and HCV Mono-Infected or Co-Infected Patients
Source: Viruses. 2022 Aug 18;14(8):1811. doi: 10.3390/v14081811 (PMC9412373; doi:10.3390/v14081811)
Supplement: Supplementary file 1 [file viruses-14-01811-s001.zip › Table S1.pdf]

Supplementary Table S1 Demographic and clinical features of 109 study participants

Variables	HCs (N=22)	HIV (N=18)	HCV (N=40)	HIV/HCV (N=29)	P value
Age, year (IQR)	54.0 (48.0,60.0)	50.0 (46.0,59.3)	57.0 (49.0,65.0)	50.0 (47.5,56.5)	0.084
Gender, n (%)					
Male	5 (22.7)	3 (16.7)	18 (45.0)	13 (44.8)	0.074
Female	17 (77.3)	15 (83.3)	22 (55.0)	16 (55.2)	
BMI (kg/m^2) (IQR)	24.1 (21.3,26.7)	25.2 (21.1,28.2)	23.0 (21.0,25.8)	21.4 (20.3,25.2)	0.165
BMR (kcal/day) (IQR)	1288.0 (1139.5,1418.8)	1296.0 (1208.5,1520.5)	1328.5 (1158.0,1583.0)	1420.0 (1159.0,1517.5)	0.850
Waist (cm) (IQR)	84.0 (76.0,93.5)	84.0 (78.8,94.3)	82.0 (74.0,93.0)	84.0 (74.5,90.0)	0.447
Hip (cm) (IQR)	95.0 (88.0,100.5)	95.0 (91.8,99.0)	92.0 (88.0,98.0)	93.0 (89.0,97.0)	0.437
WHR (IQR)	0.9 (0.8,0.9)	0.9 (0.9,1.0)	0.9 (0.8,0.9)	0.9 (0.8,0.9)	0.622
Body fat (%)(IQR)	30.3 (27.9,33.6)	33.3 (26.2,36.0)	27.7 (25.0,30.5)	25.4 (19.1,31.3)	0.011
VF (IQR)	8.0 (4.0,10.0)	7.5 (3.8,11.3)	7.0 (4.0,11.0)	7.0 (4.0,10.0)	0.759
FBS (mmol/L) (IQR)	4.6 (4.2,4.8)	4.5 (4.0,4.9)	4.5 (4.0,5.1)	4.6 (4.0,5.0)	0.994
**Liver Test Results (IQR)**					
ALT, U/L, median	20.5 (13.8,31.0)	22.0 (10.5,38.3)	43.0 (28.0,57.5)	52.0 (36.0,99.0)	<0.0001
AST, U/L, median	20.5 (15.0,28.0)	24.5 (20.0,34.3)	40.5 (27.8,50.0)	48.0 (30.5,76.0)	<0.0001
γ-GT, U/L, median	19.5 (13.0,27.0)	27.0 (17.8,90.5)	19.5 (15.0,35.3)	65.0 (36.5,114.5)	<0.0001
ALP, U/L, median	94.0 (78.0,106.3)	120.5 (88.5,161.3)	99.5 (87.0,127.0)	158.0 (114.0,195.0)	<0.0001
TBil, µmol/L	13.8 (10.9,16.9)	11.9 (9.9,16.9)	17.0 (14.6,27.1)	15.7 (10.3,19.1)	0.003
DBil, µmol/L	3.7 (2.7,5.4)	2.6 (1.6,4.4)	4.8 (3.5,6.9)	4.9 (1.7,8.4)	0.006
IBil, µmol/L	11.0 (9.0,13.4)	9.1 (7.3,13.2)	12.3 (10.3,19.7)	10.5 (8.8,12.5)	0.017

**Liver fibrosis markers (IQR)**					
FIB-4	1.2 (0.8,1.7)	1.4 (0.9,27)	2.7 (1.6,4.3)	2.2 (1.6,4.1)	<0.0001
APRI	0.3 (0.2,0.4)	0.3 (0.2,0.5)	0.7 (0.4,1.4)	0.8 (0.5,1.6)	<0.0001
GPRI	0.2 (0.1,0.4)	0.3 (0.2,0.7)	0.3 (0.2,0.7)	0.7 (0.4,1.9)	<0.0001
LSM (kPa)	6.0 (4.6,7.3)	4.5 (3.8,7.9)	7.3 (5.8,8.9)	7.9 (6.9,12.3)	<0.0001
CAP (dB/m)	234.7 (206.5,281.9)	242.8 (215.8,272.2)	228.2 (217.9,259.0)	226.2 (212.9,249.1)	0.759
**Routine Blood Test (IQR)**					
Percentage of lymphocyte (%)	32.4 (27.2,37.4)	36.2 (32.7,43.2)	36.2 (30.3,43.7)	37.2 (27.5,42.6)	0.278
Percentage of neutrophils (%)	60.3 (55.1,63.9)	56.9 (50.2,61.2)	56.0 (47.4,61.9)	55.5 (48.0,64.9)	0.272
Percentage of monocytes (%)	4.8 (4.1,5.3)	4.6 (3.8,5.2)	5.0 (4.5,5.7)	5.2 (3.9,5.8)	0.182
WBC (10^9/L)	6.1 (5.4,7.3)	4.6 (3.6,5.8)	4.9 (4.0,5.6)	5.2 (4.5,6.6)	0.012
RBC (10^{12}/L)	4.3 (3.8,5.3)	4.0 (3.6,4.4)	4.5 (4.1,5.0)	4.5 (3.9,4.8)	0.009
HGB (g/L)	133.0 (120.3,160.3)	130.5 (107.3,139.0)	144.5 (125.5,158.5)	143.0 (128.5,158.0)	0.019
PLT (10^9/L)	236.0 (164.8,251.3)	207.5 (143.3,292.3)	137.5 (94.8,182.0)	161.0 (105.0,189.5)	<0.0001
PDW (fL)	12.4 (11.6,14.0)	11.9 (9.8,14.0)	13.9 (12.3,16.3)	11.8 (10.7,13.9)	0.006
MPV (fL)	11.0 (10.5,11.9)	10.9 (9.7,12.3)	11.7 (10.9,12.9)	10.8 (10.4,11.8)	0.024
PCT (%)	0.2 (0.2,0.3)	0.2 (0.2,0.3)	0.2 (0.1,0.2)	0.2 (0.1,0.2)	<0.0001
$CD4^+T$ (%)	—	484.5 (201,693.5)	—	514.0 (462.5,629.0)	0.463

IQR, interquartile range; BMI, body mass index; BMR, basal metabolic rate; WHR, waist-to-hip ratio; VF, Visceral fat; FBS, fasting blood sugar; ALT, alanine aminotransferase; AST, aspartate aminotransferase; γ-GT, gamma-glutamyl transferase; ALP, alkaline phosphatase; TBil, total bilirubin; DBil, direct bilirubin; IBil, indirect bilirubin; LSM, Liver stiffness measurement; CAP, Controlled attenuation parameter; WBC, white blood cell count; RBC, red blood cell count; HGB, hemoglobin; PLT, platelet; PDW, platelet distribution width; MPV, mean platelet volume; PCT, procalcitonin; Data were shown as the median (IQR), n (%), n, where n is the actual number with available data. Categorical variables were analysed using Chi square test and continuous variables were analysed using Kruskal-Wallis test or Mann-Whitney test as appropriate based on number of groups.
