# Supplementary material for: Characterization of the Intestinal Fungal Microbiome in HIV and HCV Mono-Infected or Co-Infected Patients
Source: Viruses. 2022 Aug 18;14(8):1811. doi: 10.3390/v14081811 (PMC9412373; doi:10.3390/v14081811)
Supplement: Supplementary file 1 [file viruses-14-01811-s001.zip › Table S2.pdf]

Supplementary Table S2 Characteristics of sample sequencing length

Sample ID	1-100	101-200	201-300	301-400	401-500
HCs_10	0	0	53822	621	1
HCs_13	0	0	52534	63	1
HCs_16	0	0	47888	705	2
HCs_19	0	0	52464	4903	1
HCs_20	0	0	54925	1899	21
HCs_24	0	0	70176	2165	0
HCs_29	0	0	66164	3061	214
HCs_49	0	1	60007	7964	79
HCs_61	0	0	65906	762	28
HCs_68	0	0	59008	1118	246
HCs_82	0	0	55015	9008	5276
HCs_90	0	0	60672	549	0
HCs_100	0	0	59642	264	207
HCs_105	0	0	44951	11441	4
HCs_141	0	549	62121	149	0
HCs_171	0	43	69081	200	46
HCs_177	0	4938	63761	440	5
HCs_181	0	142	71340	455	7
HCs_183	0	97	49383	1033	68
HCs_190	0	782	68824	12	15
HCs_219	0	48	66784	973	19
HCs_233	0	296	73785	148	0
HIV_2	0	0	58379	36	139
HIV_6	0	1178	67004	749	16
HIV_7	0	1777	68658	395	38
HIV_8	0	26	66657	175	0
HIV_14	0	759	65726	29	0
HIV_21	0	0	56863	878	112
HIV_55	0	0	43185	457	210
HIV_62	0	0	52307	2525	22
HIV_75	0	0	52169	1852	102
HIV_121	0	0	46472	65	121
HIV_123	0	0	34092	23	227
HIV_127	0	0	49917	50	0
HIV_131	0	0	53835	412	2
HIV_147	0	0	49932	278	19
HIV_162	0	0	52913	54	203
HIV_179	0	0	57302	1102	0
HIV_210	0	23	53747	424	0
HIV_230	0	0	52286	106	0
HCV_3	0	0	49022	332	1

HCV_5	0	0	46309	1412	0
HCV_9	0	0	49475	206	0
HCV_15	0	0	46924	272	4
HCV_32	0	0	43156	489	3
HCV_35	0	0	42468	1244	5
HCV_42	0	0	49606	549	0
HCV_46	0	0	36393	2013	1
HCV_47	0	0	72150	473	4
HCV_54	0	0	46904	1109	0
HCV_58	0	0	55014	1182	0
HCV_70	0	0	47319	3581	2
HCV_73	0	0	36072	355	30
HCV_77	0	0	55360	35	0
HCV_79	0	0	47751	28	0
HCV_99	0	0	55652	1109	1
HCV_102	0	0	48067	44	0
HCV_104	0	0	44811	1469	171
HCV_111	0	0	53114	22	0
HCV_117	0	0	53676	1	0
HCV_134	0	1	52920	24	6
HCV_139	0	1	45876	5797	2
HCV_140	0	0	37759	475	67
HCV_142	0	0	57482	0	0
HCV_152	0	0	48240	175	0
HCV_153	0	0	56621	483	0
HCV_154	0	0	43235	2215	18
HCV_158	0	0	44062	1036	75
HCV_168	0	0	31187	4586	2
HCV_169	0	0	48196	221	2
HCV_178	0	0	57502	140	0
HCV_182	0	0	48035	132	8
HCV_188	0	0	55123	23	0
HCV_195	0	1	59363	200	25
HCV_196	0	10	51337	317	28
HCV_200	0	1	62395	441	0
HCV_201	0	0	61027	520	35
HCV_208	0	86	70722	1298	0
HCV_212	0	0	72591	426	1
HCV_229	0	0	48732	1922	73
HIVHCV_11	0	0	46331	233	0
HIVHCV_25	0	0	44104	3005	0
HIVHCV_26	0	44	53744	236	0
HIVHCV_28	0	10	46117	316	0

HIVHCV_36	0	0	54992	202	145
HIVHCV_38	0	0	51530	2269	150
HIVHCV_44	0	8	61042	101	6
HIVHCV_52	0	0	48793	867	14
HIVHCV_67	0	0	30178	6	0
HIVHCV_76	0	0	45533	113	40
HIVHCV_85	0	0	45895	463	0
HIVHCV_93	0	0	74072	852	0
HIVHCV_96	0	1	43008	215	0
HIVHCV_98	0	0	53627	1	0
HIVHCV_106	0	0	42770	2	0
HIVHCV_110	0	0	47678	70	1
HIVHCV_130	0	0	44234	2155	1
HIVHCV_136	0	0	49597	24	21
HIVHCV_143	0	0	52160	288	1
HIVHCV_145	0	0	48040	837	1
HIVHCV_146	0	0	50968	245	1
HIVHCV_148	0	0	71799	270	0
HIVHCV_150	0	27	48845	10	8
HIVHCV_151	0	1	70574	214	114
HIVHCV_161	0	499	60150	1021	82
HIVHCV_164	0	44	69692	145	29
HIVHCV_165	0	33	74367	53	49
HIVHCV_176	0	1821	66145	857	18
HIVHCV_194	0	345	72763	143	0
