# Supplementary material for: Characterization of the Intestinal Fungal Microbiome in HIV and HCV Mono-Infected or Co-Infected Patients
Source: Viruses. 2022 Aug 18;14(8):1811. doi: 10.3390/v14081811 (PMC9412373; doi:10.3390/v14081811)
Supplement: Supplementary file 1 [file viruses-14-01811-s001.zip › Table S3.pdf]

Supplementary Table S3 Comparison of fungal alpha diversity indexes among four groups.

Estimators	HCs-Mean±Sd	HIV-Mean±Sd	HCV-Mean±Sd	HIVHCV-Mean±Sd	$P_{HIV-HCs}$	$P_{HCV-HCs}$	$P_{HIV/HCV-HCs}$
Sobs	111.14±29.60	105.78±29.624	115.65±71.61	97.79±38.09	1.00	0.59	0.05*
Shannon	2.07±0.63	2.07±0.82	2.11±1.00	2.26±0.78	0.92	0.82	0.20
Simpson	0.29±0.17	0.30±0.21	0.32±0.26	0.25±0.20	0.11	0.62	0.19
Ace	129.08±25.96	116.62±29.89	136.96±76.71	112.59±35.57	0.21	0.48	0.02*
Chao	124.57±28.76	115.47±30.25	131.91±77.614	110.18±37.84	0.10	0.60	0.04*

Data are expressed as mean ± standard deviation, and the Wilcoxon Rank-Sum test was used for comparison between groups, and the P value less than 0.05 were significant.
