# Supplementary material for: Characterization of the Intestinal Fungal Microbiome in HIV and HCV Mono-Infected or Co-Infected Patients
Source: Viruses. 2022 Aug 18;14(8):1811. doi: 10.3390/v14081811 (PMC9412373; doi:10.3390/v14081811)
Supplement: Supplementary file 1 [file viruses-14-01811-s001.zip › Table S4.pdf]

Supplementary Table S4. Correlation among gut fungi in study participants.

Group	Fungi	Fungi	Correlation	P-value
HCs	*g__Alternaria*	*g__Fusarium*	0.6455	0.0009
	g__Acremonium	*g__Cladosporium*	0.6298	0.0013
	g__Cladosporium	*g__Simplicillium*	0.8031	<0.0001
	g__Cladosporium	*g__unclassified_k__Fungi*	0.7008	0.0002
	g__Fusarium	*g__Penicillium*	0.7458	<0.0001
	g__Simplicillium	*g__unclassified_k__Fungi*	0.6584	0.0006
	g__Gelasinospora	*g__Saccharomycopsis*	0.6323	0.0012
	g__Gelasinospora	*g__Curvularia*	0.6022	0.0024
	g__unclassified_f__Debaryomycetaceae	*g__Buckleyzyma*	0.7945	<0.0001
	g__Eremothecium	*g__unclassified_o__Saccharomycetales*	0.6022	0.0024
	g__Saccharomycopsis	*g__Curvularia*	0.6474	0.0008
	g__Ilyonectria	*g__unclassified_o__Tremellales*	0.6260	0.0014
	g__Hormographiella	*g__Kazachstania*	0.6120	0.0019
	g__Kodamaea	*g__Pichia*	0.7505	<0.0001
	g__unclassified_f__Debaryomycetaceae	*g__Pseudopithomyces*	0.6766	0.0004
	g__Kazachstania	*g__Saccharomyces*	0.6184	0.0017
	g__Buckleyzyma	*g__unclassified_o__Tremellales*	0.6823	0.0003
HIV	*g__Aspergillus*	*g__Candida*	-0.9360	<0.0001
	g__Aspergillus	*g__unclassified_o__Saccharomycetales*	-0.6390	0.0043
	g__Alternaria	*g__Didymella*	0.6897	0.0015
	g__Auricularia	*g__Epicoccum*	0.6053	0.0078
	g__Auricularia	*g__unclassified_o__Helotiales*	0.6116	0.0070
	g__Cladosporium	*g__Wallemia*	0.7186	0.0008

	g__Coprinellus	*g__Rhodotorula*	-0.6000	0.0085
	g__Coprinellus	*g__unclassified_c__Sordariomycetes*	0.6295	0.0051
	g__Chaetomium	*g__unclassified_p__Basidiomycota*	0.7244	0.0007
	g__Clavispora	*g__unclassified_o__Helotiales*	0.6420	0.0041
	g__Clavispora	*g__unclassified_p__Basidiomycota*	0.6032	0.0080
	g__Cystobasidium	*g__unclassified_c__Sordariomycetes*	0.6599	0.0029
	g__Didymella	*g__Epicoccum*	0.6132	0.0068
	g__Didymella	*g__Preussia*	-0.6262	0.0054
	g__Debaryomyces	*g__Fusarium*	0.7553	0.0003
	g__Epicoccum	*g__Preussia*	-0.6495	0.0035
	g__Preussia	*g__Thelebolus*	0.8095	<0.0001
	g__Phialophora	*g__unclassified_o__Helotiales*	0.6116	0.0070
	g__Pseudopithomyces	*g__unclassified_p__Rozellomycota*	0.7276	0.0006
	g__Talaromyces	*g__unclassified_k__Fungi*	0.6712	0.0023
	g__Tausonia	*g__unclassified_o__Saccharomycetales*	0.6803	0.0019
	g__Tausonia	*g__unclassified_f__Pleurotheciaceae*	0.6000	0.0085
	g__Tausonia	*g__unclassified_p__Basidiomycota*	0.6052	0.0078
	g__Wickerhamomyces	*g__Xeromyces*	0.6082	0.0074
	g__unclassified_o__Helotiales	*g__unclassified_f__Pleurotheciaceae*	0.7276	0.0006
HCV	*g__Aspergillus*	*g__Candida*	-0.6786	<0.0001
	g__Penicillium	*g__unclassified_k__Fungi*	0.6497	<0.0001
	g__unclassified_p__Ascomycota	*g__unclassified_k__Fungi*	0.5957	<0.0001
	g__Cladosporium	*g__unclassified_k__Fungi*	0.5928	<0.0001
	g__Cladosporium	*g__Wallemia*	0.6630	<0.0001
	g__Wallemia	*g__unclassified_k__Fungi*	0.6988	<0.0001

	g__Fusarium	*g__Gibberella*	0.5910	<0.0001
HIV/HCV	*g__Aspergillus*	*g__Candida*	-0.6207	0.0003
	g__Fusariella	*g__Olpidium*	0.8014	<0.0001
	g__Fusariella	*g__Westerdykella*	0.8014	<0.0001
	g__Olpidium	*g__Westerdykella*	1.0000	<0.0001
	g__Cladosporium	*g__Wallemia*	0.7463	<0.0001
	g__Alternaria	*g__Gibberella*	0.6347	0.0002
	g__Acremonium	*g__Rhodotorula*	0.6221	0.0003
	g__Mortierella	*g__Sarocladium*	0.6043	0.0005
	g__Mortierella	*g__unclassified_o__Sordariales*	0.7881	<0.0001
	g__Sarocladium	*g__unclassified_o__Sordariales*	0.7090	<0.0001
	g__unclassified_f__Microascaceae	*g__unclassified_o__Sordariales*	0.6788	<0.0001
