# Supplementary figures and images for: Characterization of the Intestinal Fungal Microbiome in HIV and HCV Mono-Infected or Co-Infected Patients
Source: Viruses. 2022 Aug 18;14(8):1811. doi: 10.3390/v14081811 (PMC9412373; doi:10.3390/v14081811)

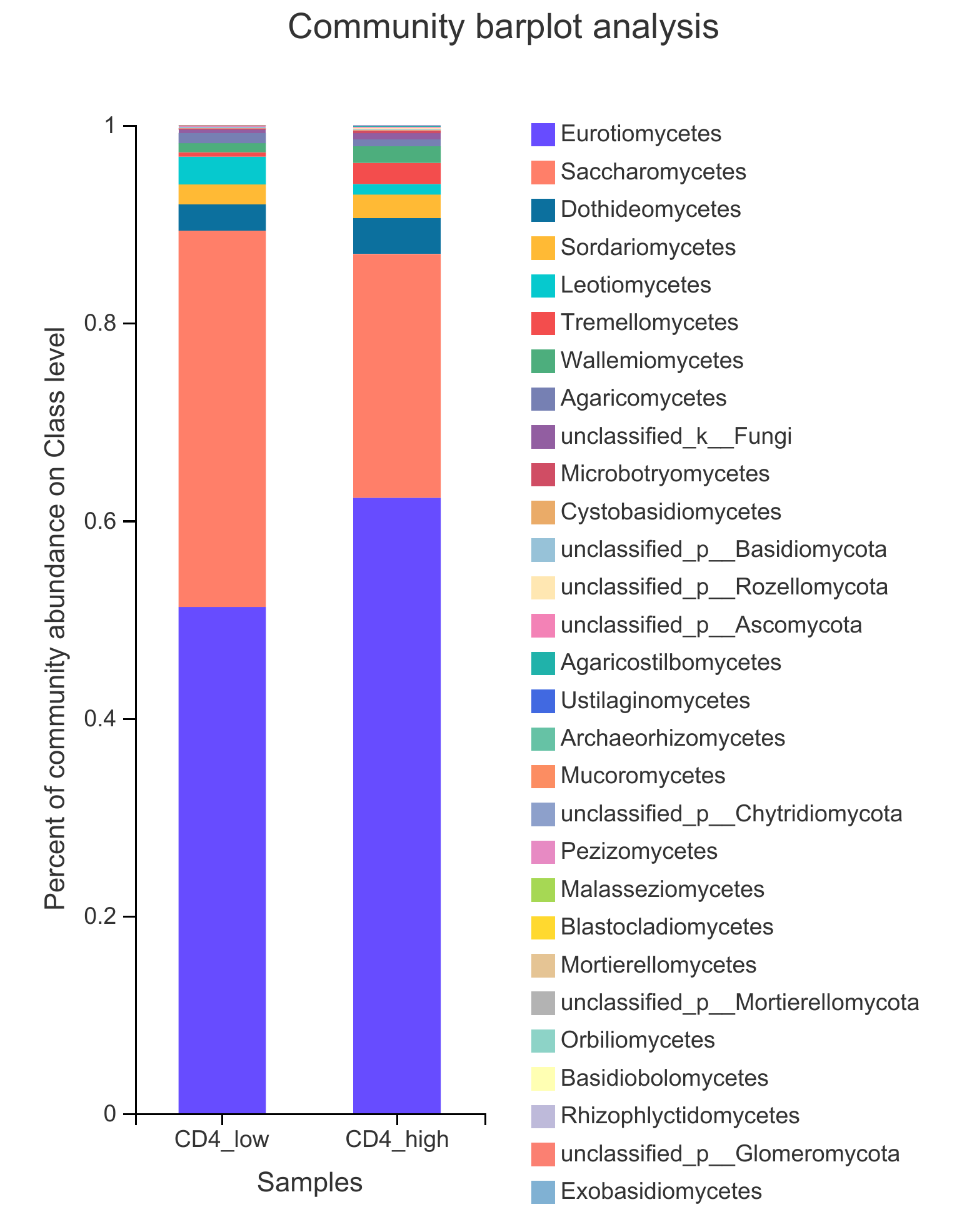

Supplement: Supplementary file 1 [file viruses-14-01811-s001.zip › Figure S2.tif]

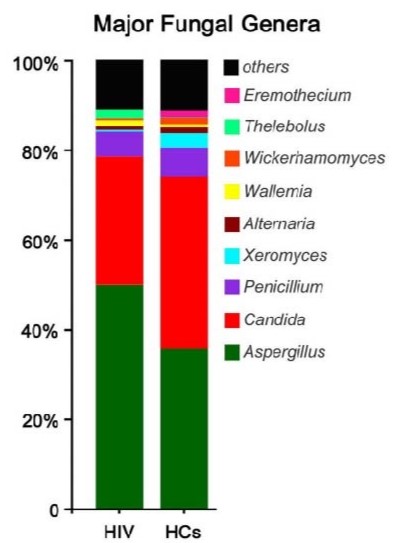

Supplement: Supplementary file 1 [file viruses-14-01811-s001.zip › FigureS1.jpg]
